# Iodine deficiency and real-life supplementation ineffectiveness in Polish pregnant women and its impact on thyroid metabolism

**DOI:** 10.3389/fendo.2023.1068418

**Published:** 2023-06-16

**Authors:** Dorota Filipowicz, Ewelina Szczepanek-Parulska, Aniceta A. Mikulska-Sauermann, Marta Karaźniewicz-Łada, Franciszek K. Główka, Krzysztof Szymanowski, Mariusz Ołtarzewski, Lutz Schomburg, Marek Ruchała

**Affiliations:** ^1^ Department of Endocrinology, Metabolism and Internal Medicine, Poznan University of Medical Sciences, Poznan, Poland; ^2^ Department of Physical Pharmacy and Pharmacokinetics, Poznan University of Medical Sciences, Poznan, Poland; ^3^ Department of Perinatology and Gynaecology, Poznan University of Medical Sciences, Poznan, Poland; ^4^ Institute of Mother and Child, Warsaw, Poland; ^5^ Institute of Experimental Endocrinology, Charité - Universitätsmedizin Berlin, Berlin, Germany

**Keywords:** urinary iodine concentration (UIC), micronutrients at pregnancy, iodine, selenium, hypothyroidism, thyroiditis, neonatal cord blood, pregnancy supplementation guidelines

## Abstract

**Introduction:**

Iodine is a pivotal component of thyroid hormones, and its deficiency leads to negative pregnancy outcomes. Therefore, during gestation, additional iodine supplementation is recommended.

**Objectives:**

By evaluating a group of women from western Poland, the study updated on iodine status during pregnancy and the effectiveness of iodine supplementation in relation to the maternal and neonatal thyroid function.

**Patients and methods:**

A total of 91 women were recruited before the delivery between 2019 and 2021. During the medical interview, the patients declared their dietary supplements intake. Thyroid parameters (TSH, ft3, ft4, a-TPO, a-Tg, and TRAb) were measured in the serum of mothers and in the cord blood of newborns after birth. Urinary iodine concentration (UIC) and urine/creatinine (UIC/crea) ratio were assessed in single urine samples using a validated high-performance liquid chromatography with ultraviolet detection (HPLC-UV). Neonatal TSH screening from dried blood spot was analyzed.

**Results:**

Pregnant women showed a median (interquartile range) UIC of 106 (69–156) µg/liter and UIC/crea ratio of 104 (62–221) µg/g, whereas approximately 20% had UIC/crea below 50 µg/g, indicating iodine deficiency. The iodine supplementation ratio was 68%. No significant differences in UIC, UIC/crea and thyroid parameters were found between iodine supplemented and non-supplemented groups; however, the highest ioduria was detected when iodine was supplemented in addition to levothyroxine in comparison with both substances administered separately. Patients with UIC/crea within 150–249 µg/g demonstrated the lowest TSH and a-TPO levels. Screening TSH was above 5 mIU/liter in 6% of children.

**Conclusions:**

Despite the national salt iodization and the recommendation to supplement iodine during gestation, the status of the abovementioned microelement and real-life intake revealed the ineffectiveness of the current iodine-deficiency prophylaxis model in pregnancy.

## 1 Introduction

Thyroid dysregulation is a prevalent clinical issue, particularly relevant during pregnancy, affecting almost one-fifth of women. In the course of gestation, the requirement for thyroid hormones (THs) increases by 25–50%, due to the elevation in thyroid-binding globulin production, placental transfer of iodine and thyroxine (T4) to the fetus, TH degradation in the placenta and the enhanced renal microelement clearance (1). Moreover, THs are involved in the neuronal migration and myelination during fetal nervous system development (2). Notably, iodine represents a fundamental component of THs. Pregnant women with iodine deficiency frequently suffer from hypothyroidism (HT) as well as goiter and present an increased risk of developing thyroid autoimmunity (3). Moreover, TH deficiency results in poorer obstetric outcomes, such as higher prevalence of miscarriages, stillbirths, growth retardation, and congenital abnormalities (4). Significant iodine deficiency in prenatal life leads to the most severe form of HT, whereas mild or moderate maternal lack of iodine may cause intellectual disability, disturbed psychomotor function, poorer socialization, and a decreased IQ score in school-aged children (5, 6). In 1997 in Poland, the iodine deficiency prophylaxis program was introduced, involving mandatory iodization of household salt [20–40 mg potassium iodide (KI) per kg of salt] or neonate formulas (10 μg/100 ml of milk), and recommending additional supplementation of 100–150 μg KI per day for pregnant and breastfeeding women, which subsequently restored the optimal iodine status in the general population (7). In terms of epidemiology, it eradicated the endemic goiter in children and reduced its prevalence in pregnant women (from 80 to 19%), decreased neonatal transient HT (from 2 to 0.16%), as well as limited the thyroid cancer incidence in women over 40 years of age (8, 9). According to the latest WHO report, Poland fulfilled the requirement of > 90% of households with iodinated salt (7).

However, salt consumption may currently decrease due to the high incidence of hypertension in the population and the recommendations of the Polish Society of Hypertension, which aim to decrease the daily salt intake to 5 g/day or due to a higher proportion of imported food in the local diet (10, 11). Nevertheless, in terms of the optimal ioduria, following a 10- and almost 20-year follow-up after the introduction of the prophylaxis, the program has undoubtedly failed with regard to pregnant and lactating women (12, 13). Therefore, the necessity of additional iodine supplementation in the dose of 150–200 µg daily during pregnancy and the lactation period was particularly emphasized in the latest guidelines of the Polish Society of Endocrinology (14).

In contrast, according to the consensus of the World Health Organization (WHO), the United Nations Children’s Fund (UNICEF) and the International Council for Control of Iodine Deficiency Disorders (ICCIDD), currently renamed as the Iodine Global Network (IGN), iodine supplementation is not recommended when the general population has been iodine-sufficient over the previous 2 years, as expressed by urinary iodine concentration (UIC) >/= 100 µg/liter, whereas the UIC value of < 150 µg has been defined as a deficiency in the course of pregnancy (15). The abovementioned recommendations are ambiguous, although they remain unanimous regarding the benefits of continuous monitoring and updating iodine status in pregnancy worldwide. The available national data evaluating iodine deficiency prophylaxis effectiveness were obtained from central, northern, and southern regions of Poland, in which all experts concur regarding the ineffectiveness of this model among pregnant women. However, to date, no studies have been conducted on residents of the western part of the country (12, 16–18).

The objective of the study was to evaluate iodine status in pregnant women and assess the supplementation rate, effects, and guidelines adherence in a real-life setting among pregnant women from western Poland, with a particular emphasis on maternal and neonatal thyroid function.

## 2 Patients and methods

The study comprised 91 Caucasian women from the area of Greater Poland (western region of Poland) who were enrolled on admission to the obstetric ward prior to their term delivery. They were randomly recruited in a public hospital, which is the leading obstetrics center in the western Poland—Gynecological and Obstetric Clinical Hospital at Poznan University of Medical Sciences (tertiary referral center) within the period between 2019 and 2021. The group consisted of women, who were healthy, euthyroid, or hypothyroid in the range adjusted to the pregnancy status, with or without antithyroid antibodies [anti-thyroid peroxidase (a-TPO), anti-thyroglobulin (a-Tg), anti–TSH-receptor (TRAb)], and either treated or not with levothyroxine (LT4). They were divided into subgroups receiving supplementation or not, treated with LT4 or not, as well as into a subgroup with or without antithyroid antibodies. Patients were in good general condition, with a negative history of any serious chronic diseases, malignancies, or renal/liver disease (except for benign cysts). None of the patients declared a specific type of diet, including a fish-rich, vegan, or vegetarian diet, and the patients denied foreign trips lasting more than 1 month.

Patients were screened and interviewed by instructed midwives in terms of inclusion/exclusion criteria and the intake of dietary supplements during pregnancy, including product brand name, dose, frequency, and duration. A random single spot urine sample was collected before the delivery and stored in a freezer at −20°C in the amount of approximately 10 ml. Additionally, a maternal non-fasting venous blood sample was taken by venipuncture prior to the delivery. Up to 2 ml of cord blood from the neonatal part of the placenta was collected during the third phase of labor after cessation of umbilical cord pulsation. Both serum samples were stored frozen in −20°C. Ioduria, as UIC, was measured using a validated ion-pair high-performance liquid chromatography with ultraviolet detection (HPLC–UV), as described (19). Creatinine was determined in the same urine sample by a colorimetric enzyme-linked immunosorbent assay (ELISA) detection kit (ThermoFisher, EIACUN, Frederick, USA). In order to objectify the results, the urinary iodine/creatinine ratio (UIC/crea) was calculated. UIC and UIC/crea results were analyzed according to the latest WHO criteria for iodine status assessment in pregnant women (20). Concentrations of serum TSH, ft3, ft4, and antithyroid antibodies (a-TPO, a-Tg) were measured by means of electrochemiluminescence (ECLIA, Hitachi and Roche Diagnostics kits) using Cobas e601 analyzer (Indianapolis, IN, USA) and TRAb by radioimmunoassay (RIA, BRAHMS Diagnostics, Berlin, Germany). Neonatal TSH was verified on the 3rd–4th day of life (TSHs) as part of national screening for congenital HT. The measurement was performed in dried blood spots from the heel puncture by an immunoluminometric (LIA) assay.

The study was performed in accordance with the Declaration of Helsinki (21) and was approved by The Local Bioethics Committee of Poznan University of Medical Sciences (protocol no. 104/19, date of approval: 10 January 2019, annexed 4 February 2021, protocol no. 132/21).

### 2.1 Statistical analysis

Statistica, version 13.3 (TIBCO Software Inc., California, USA), GraphPad Prism, version 9.5.1. (GraphPad Software, LCC, Boston, USA) and Microsoft Excel (2019) from Microsoft Office (Adobe Inc., California, USA) were used for statistical calculations. The data were not normally distributed, according to Shapiro–Wilk’s test, thus non-parametric statistical tests were applied. The groups were compared using the Mann–Whitney *U* or the paired Wilcoxon test (when parameters were analyzed within the mother–child pairs), as well as the analysis of variance (ANOVA)/Kruskal–Wallis tests with *post-hoc* analysis. The Spearman R test was performed to analyze correlations between the parameters. The results are presented mainly as the median and interquartile range (IQR, Q1–Q3), 95% confidence intervals (95% CIs), or mean with standard deviation (± SD). A *p*-value < 0.05 was considered significant.

## 3 Results

The patients’ characteristics are presented in **Table 1**.

**Table 1:** Clinical and biochemical characteristics of the recruited women and newborns.

Clinical parameter (median, IQR)	Mothers [*n* = 91]	Newborns [*n* = 101] ^b^
Age, years	33 (31–35)	
Body weight before delivery, kg	62 (56–70)	
Pregnancy week at delivery	39 (38–40)	
Body weight at delivery, g		3415 (3100–3650)
5th minute Apgar score		10 (10–10)
LT4 treated/non-treated, number of patients	50/41	
LT4 dose in 3rd trimester, µg/24h	75 (50–91)	
AIT [+]/AIT [−], number of patients	18/71	

Biochemical parameter (median, IQR)	Mothers [*n* = 89]	Newborns [*n* = 94]
TSH from venous/cord blood, µU/ml	1.99 (1.32–2.81)	8.53 (5.91–11.2)
TSHs, mIU/liter [*n* = 101]		1.88 (0.63–2.78)
ft3, pmol/liter	4.05 (3.64–4.38)	2.05 (1.78–2.35)
ft4, pmol/liter	13.7 (12.2–15.5)	16.4 (15.1–17.6)
a-TPO, IU/ml	12 (10–20)	9 (9–12)
a-Tg, IU/ml	13 (11–17)	13 (10–15)

LT4, levothyroxine; AIT, autoimmune thyroiditis: present [+], absent [−]; IQR, interquartile range; TSH, thyrotropin; TSHs, TSH at 3rd–4th day of life; ft3, free triiodothyronine; ft4, free thyroxine; a-TPO, anti-thyroid peroxidase; a-Tg, anti-thyroglobulin; ^b^ data comprises 10 twin pregnancies.

Pregnant women from the western Poland showed a median (IQR) UIC 106 (69–156) µg/liter and UIC/crea ratio equal to 104 (62–221) µg/g, which is defined as iodine deficiency (UIC or UIC/crea < 150 µg/liter or µg/g) for this particular group, according to the WHO ranges (15); see **Table 2**. Approximately 20% had UIC/crea below 50 µg/g, which is a borderline value for another indicator of iodine deficits. The correlation was observed between both indicators of ioduria used in the study, that is, UIC and UIC/crea (*R* = 0.63, *p* < 0.001).

**Table 2:** Median ioduria of pregnant women according to the WHO classification.

Category of iodine supply in population of pregnant women	WHO criteria for ioduria, expressed by a median UIC, µg/liter or UIC/crea, µg/g
Severe deficiency	< 50
Insufficient	< 150
Sufficient	150–249
Above requirements	250–499
Excessive	> 500

WHO, World Health Organization; UIC, urinary iodine concentration; UIC/crea, iodine/creatinine ratio.

Supplementation of iodine during pregnancy was declared by 62/91 pregnant women (68%). In the majority of cases, iodine was ingested as a part of multivitamin diet supplements in a median dose (IQR) of 200 (150–200) per day, with a range extending from 100–300 µg/day. In 13 out of 62 (21%) cases, iodine was not supplemented throughout the entire pregnancy, 4/62 (6.5%) of patients administered it only in the 1st and 2nd trimester, and 9/62 (14.5%) only in the 3rd trimester of pregnancy. The dose and formula of the dietary supplements are presented in **Table 3**.

**Table 3:** Iodine supplements, doses, and formulas declared by pregnant women.

Percentage of women on iodine supplements, number from total [*n* = 62]	Formula (multivitamin supplements of diet)	Dose, µg/per day
63% (39)	KIO_3_, KI	200
31% (19)	KI	150
3% (2)	KI	220/300
1.5% (1)	KI	100
1.5% (1)	Not known	Not known

KIO_3_, potassium iodate; KI, potassium iodide.

In the subsequent stages of the analysis, women who did not supplement iodine in the 3rd trimester and patients who presented abnormal TSH for this period of pregnancy were excluded.

**Figure 1:**
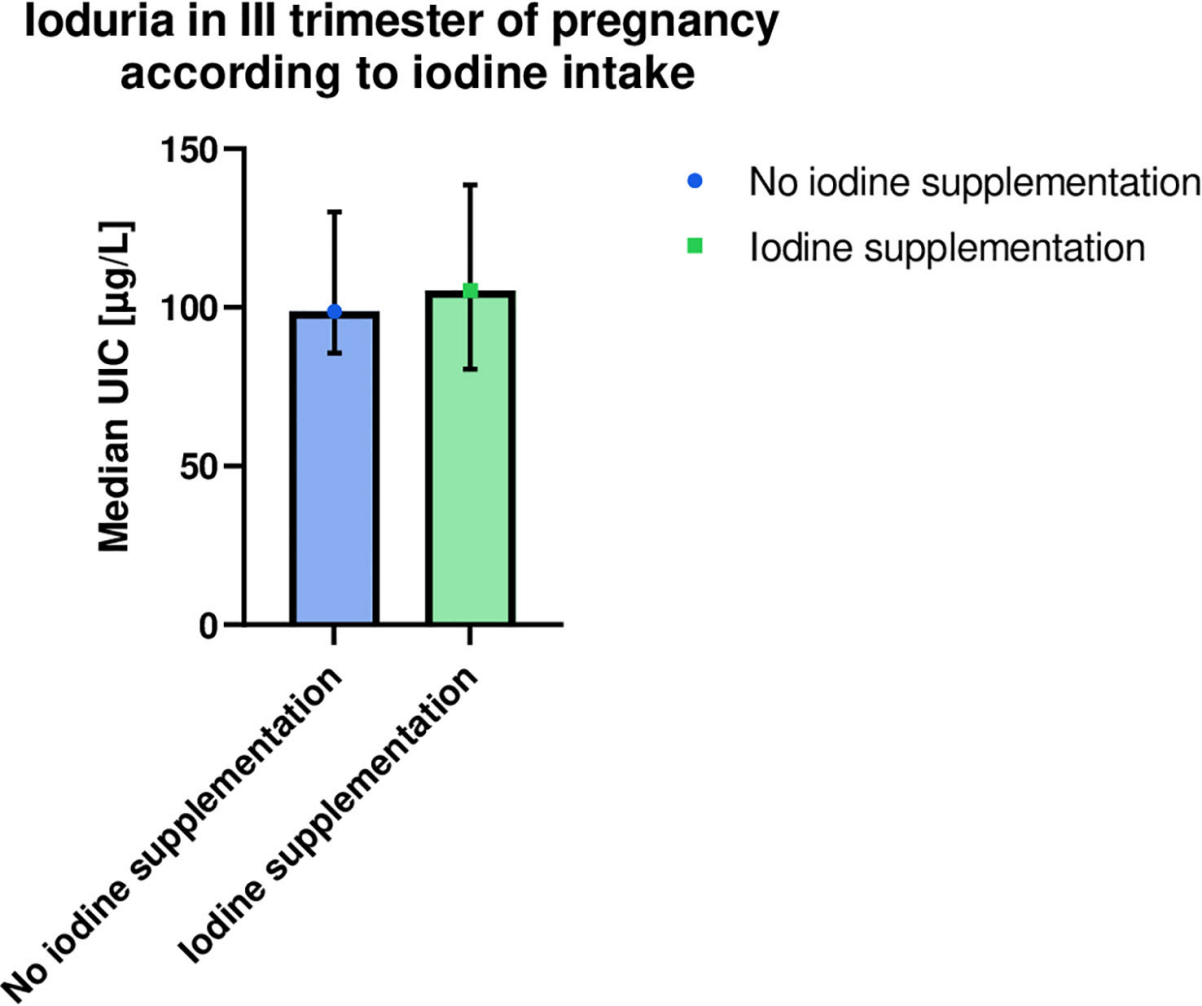
Comparison of ioduria in the last trimester of pregnancy in women who confirmed or denied iodine supplementation. Higher median UIC and wider confidence interval (95% CI) were observed in the iodine supplementing group, although the result was not significant (*p* = 0.55). Both intervals are below the normal range established by the World Health Organization (< 150 μg/liter). Medians (symbols), 95% CI (error bars). UIC, urinary iodine concentration.

No significant differences in UIC (median, Q1–Q3) were found in the group which declared iodine supplementation (105 µg/liter, 69–170, *n* = 50) in comparison with the women who denied iodine supplementation (99 µg/liter, 84–130, *n* = 27) during pregnancy (*p* = 0.55); see **Figure 1**.

There was no significant difference in UIC between the iodine-supplemented and non-supplemented groups, also in the subgroups of patients not treated with LT4 (*p* = 0.47) as well as those receiving the medication (*p* = 0.15).

No difference was found between the group of iodine-supplementing and non-supplementing mothers and those showing UIC below and above 150 µg/liter in maternal and neonatal thyroid hormone indices (TSH, ft3, ft4, and TSHs), even after excluding patients receiving LT4. There were also no differences in maternal and child antibodies (a-TPO, a-Tg, and TRAb) with regards to iodine supplementation, tested in both AIT(+) and AIT (−) subgroups.

All thyroid antibodies of mothers and their children correlated positively (a-TPO with *p* < 0.001, *R* = 0.59; a-Tg with *p* < 0.001, *R* = 0.52; TRAb with *p* < 0.001, *R* = 0.49). Additionally, the children’s ft4 correlated with maternal TRAb level (*p* = 0.03, *R* = −0.26), and cord blood TSH correlated with the mothers’ UIC (*p* = 0.03, *R* = 0.25).

**Figure 2:**
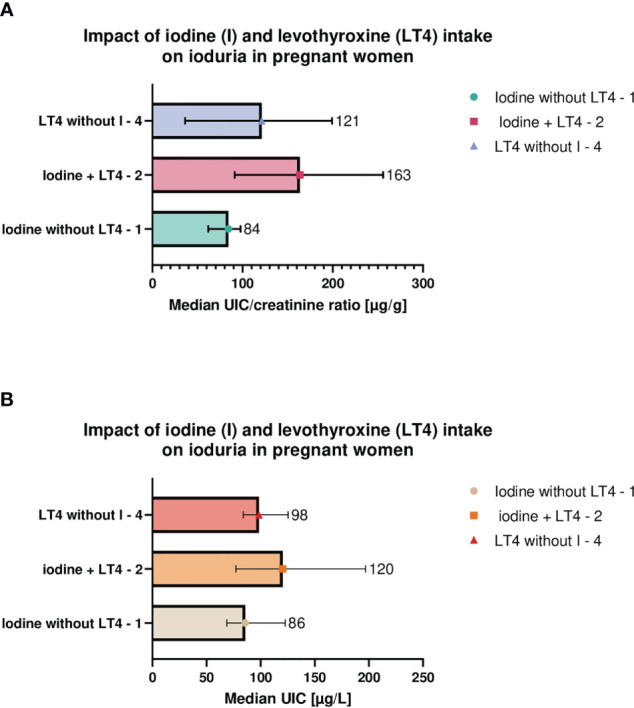
Maternal urinary iodine concentration **(A)** and iodine/creatinine ratio **(B)** in relation to levothyroxine and/or iodine supplements intake. Pregnant women differed with respect to iodine **(I)** and levothyroxine (LT4) intake and were divided into three groups; I only (group 1), LT4 only (group 2), or the combined I and LT4 intake (group 3). No significant differences in urinary iodine concentration (UIC, *p* = 0.18, KW-H = 3.46) and iodine/creatinine ratio (UIC/crea, *p* = 0.09, KW-H = 5.84) between the groups were observed. However, the highest UIC or UIC/crea were observed in the group with the combined LT4 therapy and I supplementation. Medians (symbols and numbers), 95% CI (error bars) are shown. UIC/crea, urine/creatinine ratio; LT4, levothyroxine; I, iodine; KW-H, the result of Kruskal–Wallis test.

A comparison of the three groups of women receiving external iodine source, that is, iodine without LT4, LT4 without iodine and the combined iodine and LT4 intake, revealed no significant differences regarding UIC (*p* = 0.18) or UIC/crea (*p* = 0.09). However, the highest ioduria was reported in the group with the combined LT4 and iodine intake; see **Figure 2**.

Women with UIC/crea within 150–249 µg/g presented the lowest values of TSH (in the subgroup after excluding LT4-treated patients) and a-TPO (in the subgroup with the negative antithyroid antibodies) in comparison with those with a lower or higher ioduria (**Table 4**).

**Table 4:** The impact of maternal ioduria (according to the WHO criteria) on thyroid parameters.

	UIC/crea, µg/g			H	P
	< 150	150–249	> 249		
M UIC/crea, µg/g [Me, Q1–Q3]	64.9 ^c,d^ [35.7–91.2]	182.8 ^c^ [164.3–203.5]	348.2 ^d^ [271–457.1]	60.4	< 0.01
M TSH, µU/ml [Mean, SD]*	2.06 ± 0.82 ^b^	1.42 ± 0.69 ^b^	1.97 ± 0.73	7.9	0.02
M a-TPO, IU/ml [Me, Q1–Q3]**	11^e^ [10–13.5]	10 ^a,e^ [9–11]	13 ^a^ [10.5–16]	7.96	0.02

UIC/crea, iodine/creatinine ratio; Me, median; H, the result of Kruskal–Wallis test; P, level of significance for Kruskal–Wallis test; Q1, first quartile; Q3, third quartile; M, mothers; TSH, thyrotropin; a-TPO, anti-thyroid peroxidase; post-hoc analysis revealed significant differences between the labeled subgroups with ^a^p = 0.025, ^b^p = 0.014, ^c^p < 0.001, ^d^p < 0.001, ^e^p = 0.063; *analysis among patients not treated with levothyroxine; **analysis in the group of patients with excluded elevated antithyroid antibodies levels.

Additionally, TSHs was above 5 mIU/liter in 6% of newborns. All newborns showed TSH < 12 mIU/liter (one had > 10 mIU/liter); thus, none of the children required further investigation for congenital HT.

## 4 Discussion

Our study aimed to characterize the iodine status of pregnant women in the leading obstetric center in western Poland, as well as to analyze whether supplementation involving regular nutritional supplement preparations was effective in correcting iodine deficiency. The data indicate that the presented group of pregnant women residing in the area of western Poland is iodine deficient, despite the fact that the entire country is considered to be optimally iodine-supplied at the population level. Approximately one-third of pregnant women denied supplementing iodine in the course of pregnancy, and many supplemented the microelement inappropriately with regard to the onset and duration of the supplementation, disregarding both Polish and international guidelines.

Despite the differences in UIC among the supplemented and non-supplemented subgroups of women found in the literature, no study has shown sufficient iodine concentrations in pregnant Polish women with regard to the UIC reference ranges established by the WHO (see **Table 5**), with the exception of the data provided in one interventional study by Jastrzębska et al. (22). In the presented study, the supplemented group did not show a significantly higher UIC, although the subjects declared taking a proper iodine dose. The abovementioned notion is in line with the observation that the supplementation rate was one of the highest in comparison with the previous Polish reports, yet it still did not significantly affect the overall ioduria. This may be attributed to inappropriate formulas used by the patients, whereby in our study only one participant received KI in the form of the prescription drug, and the rest used a multi-nutrient over-the-counter pregnancy dietary supplements. In addition, iodine supplementation was frequently initiated too late or discontinued too early (21% of subjects in our cohort received iodine only through a short period). This unfortunate inconsequence of supplementation, was already observed in the previous analyses (23). The only report where borderline sufficient ioduria was achieved, was an interventional study involving the administration of 150 μg of KI per day in a separate tablet (22). In terms of dietary supplements, KI is the most recommended form, whereas Kelp should be avoided, due to high-dose variation (24). Interestingly, the U.S. market analysis revealed that 24% of prenatal multivitamin supplements did not contain iodine at all, and in those containing iodine, the doses varied considerably (25–290 μg), with still others contained Kelp (25).

**Table 5:** A comparison of the Polish studies with regard to pregnancy ioduria in the past 14 years.

Author and type of the study	Year	Region of Poland	Number of women	Trimester	Median UIC, µg/liter	Amount of women with UIC ≥ 150 μg/liter	Neonatal TSH > 5 mIU/liter on 3rd day of life	Method of UIC measurement	Iodine supplemented (supplementation rate) *vs.* non-supplemented
D Filipowicz, (2022) [cross-sectional]	2019–2021	Western (Poznań)	91	3rd	106	28.5%	6%	HPLC–UV (spot urine sample)	105 (68%) *vs.* 99, *p* = 0.55
M Trofimiuk-Müldner, (2020) (13) [cross-sectional]	2017	Northern, southern, central, north-eastern, south-eastern	300	1st (14.7%) 2nd (21%) 3rd (64.7%)	112	–	–	Sandell–Kolthoff reaction (spot urine sample)	–
H Jastrzębska, (2016) (22) [prospective]	2008–2013	Central (Warsaw)	92	1st	1st - 83 3rd - 101	–	8.77%	Sandell–Kolthoff reaction (spot urine sample)	151.5 (100% in 2nd and 3rd) *vs.* 101, *p* < 0.01
M Krasnodebska-Kiljańska, (2013) (17) [prospective]	–	Central (Warsaw)	62	1st	1st - 96 2nd - 122 3rd - 129	14%	4.41%	–	(100% in 2nd and 3rd)
A Zygmunt, (2015) (18) [cross-sectional]	2010	Central (Łódź)	115	1st (6%) 2nd (53%) 3rd (41%)	1st - 80.1 2nd - 81.3 3rd - 78.4	–	2%	Sandell–Kolthoff reaction (spot urine sample)	129.4 (45%, 1st - 0, 2nd - 24.6%, 3rd - 78.7%) *vs.* 73.0, *p* < 0.001
**M Gietka-Czernel, (2010)** (12) **[cross-sectional]**	2007-2008	Central (Warsaw)	100 (72)	1st (32%) 2nd (36%) 3rd (32%)	112.6	28%	2.9%	Sandell–Kolthoff reaction (24h urine collection)	146.9 (35%, 1st - 31%, 2nd - 39%, 3rd - 34%) *vs.* 97.3, *p* < 0.001
T Milewicz, (2011) (16) [cross-sectional, survey]	–	Southern (Kraków)	500	–	–	–	–	–	(59%)

UIC, urinary iodine concentration; TSH, thyrotropin; HPLC-UV, high-performance liquid chromatography with ultraviolet detection.

Furthermore, the study group had already been evaluated with regard to the selenium intake, where the authors found both poor selenium status and a similar ineffectiveness of self-administered micronutrient supplementation (26). A similar observation was made in Latvia where, despite a wide supplement usage (70%), pregnant women failed to achieve optimal selenium and iodine status. However, the baseline concentrations of both trace elements were higher than in Poland (26). Therefore, local guidelines concerning microelement and vitamin supplementation are necessary to provide the evidence-based medicine background for physicians and self-reported users, with particular emphasis on the most beneficial groups, such as pregnant women and autoimmune thyroiditis patients (27, 28). In other European countries, where salt iodization had been introduced, the optimal ioduria was achieved in school-aged children, although not in pregnant women (29, 30). Similar results were obtained in Portuguese pregnant women in the 1st trimester, where median UIC was 104 μg/liter, 19% had UIC < 50 μg/liter, and the supplementation rate was 57%, despite a decade long official recommendations regarding iodine supplementation during pregnancy, thus, showing poor adherence to the guidelines (31). The low iodine status was associated with poorer knowledge in terms of iodine significance and its sources (32). This may also potentially account for the results obtained in the presented study.

Notably, our cohort included also hypothyroid patients, some of whom were treated with LT4, which releases 64% of iodine itself. Possibly, the local population, or individuals with a disturbed thyroid function, experienced excessive local tissue iodine deficits in the thyroid gland, or the need for this micronutrient is too great to be effectively supplemented with the recommended dose. Nevertheless, in our further calculations, we excluded the hypothyroid patients with abnormal TSH, based on the population-adjusted referenced values, established by the Polish Society of Endocrinology for the 3rd trimester of pregnancy (TSH 0.11–3.53). Additionally, bearing in mind the effect of iodine supplementation on thyroid function, in calculations comprising thyroid parameters, only women not receiving LT4 therapy were included, and accounted for the presence or absence of AIT.

Only women in the 3rd trimester of pregnancy were enrolled in this study (at one time point—a few days before the delivery, following hospital admission) in order to avoid inconsistency due to changes in UIC during pregnancy, where increased urine volume may lead to a decrease in iodine concentration in comparison with the 1st trimester (33). This may account for the lower value of UIC in this study in comparison with research including also the 1st and 2nd trimesters of pregnancy. Nevertheless, it should be emphasized that the second half of the 1st trimester is the most crucial time for the newborn’s maternal iodine intake, as the placenta is the only source of supplying a sufficient amount of T3 for the development of the fetal central nervous system until midgestation (2).

Conversely, elevated population ioduria may be harmful when median UIC exceeds 500 µg/liter, or the daily intake increases above 500 µg (twofolds higher than recommended). Elevated iodine may serve as an endocrine disruptor, causing oxidative damage to lipid membranes, disturbing thyroid hormones metabolism, or enhancing autoimmune processes, particularly in vulnerable individuals with preexisting iodine deficiencies (34). It is vital to note that, according to one report, iodine and selenium content in multivitamin supplements exceeds the amount stated on the label by the manufacturer, by up to 25% (35). Therefore, it is essential to avoid overdosing, or ingesting unknown microelements dosages, as well as poorly characterized supplement mixtures.

According to the WHO, iodine sufficiency at pregnancy, expressed by ioduria, can be estimated only on the population level (due to the significant intra- and interindividual differences). It reflects the total iodine consumption within the past few days. The median UIC values between 150 and 249 µg/liter are considered an optimal supply. As iodine is excreted in > 90% by kidneys, UIC in a spot urine sample is a biomarker of iodine status during gestation in epidemiological studies, recommended by the WHO (36). Nonetheless, in the course of pregnancy, physiological increase in renal filtration and urine iodine dilution may result in underestimating iodine concentration level. Moreover, hydration status and day-to-day iodine concentration differences may also impact the results. Hence, a reasonable additional indicator seems to be UIC/crea ratio, which reduces the impact of changes in urine volume. In pregnant women, this biomarker correlates with 24h urine iodine collection, and is consistent with serum concentration during pregnancy (33). Bearing in mind the aforementioned, in this study, both biomarkers were assessed. Additionally, for the purpose of the presented study, a new inexpensive and selective ion-pair HPLC-UV technique for iodine assessment in human urine was developed and validated. In comparison with the former method, inductively coupled plasma mass spectrometry (ICP–MS), HPLC-UV is less complicated and does not require any advanced equipment. Compared with the spectrophotometric method, using the Sandell–Kolthoff reaction (S–K), HPLC-UV does not involve the ingestion of the initial material, or the use of noxious substances (arsenic and cerium) (19, 37). Moreover, HPLC-UV provides better selectivity of the analytes to be determined in such a complex biological matrix as urine.

The impact of iodine intake on the thyroid may be reflected by the lowest TSH and a-TPO of mothers in the subgroup where ioduria was within the recommended ranges. Analogously, the Chinese cohort of more than a thousand pregnant women revealed lower TSH in the group with UIC 150–249 μg/liter, than in those with UIC 250 μg/liter or above, and estimated the 2.5 higher risk for developing subclinical HT in late pregnancy for women with UIC lower than 100 μg/liter in the 1st trimester (38). Two randomized controlled trials demonstrated a lower increase in TSH throughout pregnancy when supplemented with 200–225 µg iodine/day and in one study, and a lower maternal TSH was found at the 1st trimester in the supplemented group (39, 40). Additionally, in our study, a weak-positive correlation was observed between maternal UIC and neonatal TSH from the cord blood. However, according to meta-analyses, in the majority of studies, iodine supplementation itself did not impact maternal TSH, ft4, a-TPO and neonatal TSH (41). Low UIC (< 100 μg/liter) was found as an independent risk factor for positive a-Tg among iodine-sufficient population (42). Our research indicated that the lowest a-TPO was seen in the subgroup with the optimal ioduria. In terms of the reluctance to supplement iodine in pregnant women suffering from AIT, due to the fact that excessive iodine is considered a trigger of antithyroid autoimmunity, no significant increase in a-TPO was found after administrating a dose of 100 or 150 μg daily (43), and the prevalence of a-TPO positivity among Iranian pregnant women did not increase 2 years following introduction of a national iodine supplementation (44). Notably, in our study, a positive correlation was found between all maternal and newborns’ thyroid autoantibodies, which proved maternofetal transplacental transfer of IgG antibodies, since a neonate is unable to produce antibodies during the first months of life. Moreover, higher maternal TRAb concentration was related to a lower fetal ft4 level, which may be attributed to the presence of TSH receptor blocking autoantibodies fraction, detected by TRAb assessment, which reduce fetal thyroid hormones production.

Neonatal TSHs evaluated in a dried whole blood sample (heel prick) 3 to 4 days after birth may serve as a sensitive indicator of population iodine deficiency. The latter is recognized when TSH is above 5 mlU/liter in more than 3% of neonates, as presented in this study (20, 45). Newborns poorly supplied in iodine showed an elevated thyroidal iodine turnover. As a consequence, TSH increases in the first weeks of life, causing neonatal transient hyperthyrotropinemia. This condition resolves spontaneously after 2 weeks and should be distinguished from the physiological TSH elevation in the first 36h of life due to perinatal stress. Nevertheless, neonatal transient hyperthyrotropinemia increases the risk of developing persistent hyperthyrotropinemia in childhood (46). In the presented study, TSH elevation in the first minutes of life was verified after 3 days, and achieved physiological concentrations in all newborns, showing no correlations with former TSH results. Therefore, it should be emphasized that only a delayed TSH assessment should be taken into account.

The American Thyroid Association guidelines recommend supplementation of 150-µg iodine daily during pregnancy and lactation, although the optimal ioduria was demonstrated in this geographical area (47). In contrast to the Polish guidelines in hypothyroid women treated with LT4, they discourage additional supplementation of iodine. In the present study, among patients treated with LT4, supplementing only iodine or receiving both LT4 and iodine, no significant difference was found. However, it is worth bearing in mind that the addition of iodine to LT4 in hypothyroid patients may improve the iodine status to a greater extent than iodine or LT4 alone, which would favor local recommendations (see **Table 6**). This is supported by another Polish study, where optimal UIC was achieved after the addition of 150 μg of KI to the standard LT4 dose (22). Nevertheless, more studies are necessary to prove the currently observed tendency.

**Table 6:** Summary of the recommendations for iodine supplementation in pre-conception, pregnancy and the lactation period.

Author and publication year	Guideline title	Iodine dose per day and the formulation	Time of supplementation initiation and duration	The recommendation in case of thyroid disturbances
A Hubalewska-Dydejczyk, (2021) (14)	Thyroid diseases in pregnancy: Guidelines of the Polish Society of Endocrinology	150–200 μg of iodine or 400 mg of iodized oil per year	Pregnancy planning (50 μg), pregnant and breastfeeding	150 µg/day in mild hypothyroidism treated with a low dose of LT4 ^b^, lower iodine dose in high dose of LT4
M Zimmer, (2020) (48)	Polish Society of Gynecologists and Obstetricians recommendations on supplementation during pregnancy	150–200 μg	Pregnancy	Supplementation under control of thyroid hormones and antithyroid antibodies level
EK Alexander, (2017) (24)	2017 Guidelines of the American Thyroid Association for the Diagnosis and Management of Thyroid Disease During Pregnancy and the Postpartum	150 μg of potassium iodide or 400 mg of iodized oil per year	3 months before pregnancy, pregnancy and lactation	No supplementation in hyperthyroidism and in hypothyroidism treated with LT4 ^a^
L De Groot, (2012) (49)	Management of Thyroid Dysfunction during Pregnancy and Postpartum: An Endocrine Society Clinical Practice Guideline	150–200 μg of potassium iodide or iodate	Before conception, pregnancy and lactation	–
J H Lazarus (2014) (50)	2014 European thyroid association guidelines for the management of subclinical hypothyroidism in pregnancy and in children	150 μg of potassium iodide	Before conception, pregnancy and lactation	Need for studies on the efficacy and side effects of combined LT4 and iodine or iodine alone in subclinical hypothyroidism

LT4, levothyroxine; ^a^weak recommendation, low-quality evidence; ^b^strong recommendation, low-quality evidence.

The conducted study also has a few limitations. Median ioduria was assessed in the heterogenous group in terms of the thyroid status (including euthyroid, hypothyroid women, with and without AIT, patients receiving LT4). The data concerning supplement intake were interview-based; thus, it is impossible to exclude the bias of underreporting. Additionally, no detailed daily food questionnaire was performed; hence, the impact of individual diets, possibly rich in iodine, was also not addressed. However, any specific diet followers and individuals residing abroad for longer periods were excluded, and due to obligatory salt iodization, the population baseline iodine status was assumed to be nearly equal. Iodine was assessed only in the 3rd trimester of pregnancy, where the most complete iodine status would have been documented, with the assessments performed at least once per trimester, including the most crucial for the neonatal development—the 1st trimester.

In most countries, universal salt iodization programs are ineffective in restoring adequate maternal ioduria with regard to the ranges established by the WHO. Despite the recommendation to additionally supplement iodine during pregnancy, the real-life assessment of this trace element supplementation revealed the ineffectiveness of the current model. An interventional study would presumably need to be conducted in order to verify the effectiveness of the dose and avoid a possible bias due to self-reporting supplementation, irregularity in the supplement intake (particularly in the end of pregnancy), HT, or LT4 impact, as well as diversity of the preparations declared. Physicians should consider prescribing KI as a medication (verified composition), and it should possibly come as a separate formulation. It is essential to increase the awareness of endocrinologists, gynecologists, general practitioners, and the society, especially pregnant women, regarding the significance and the benefits of a proper iodine supplementation during pregnancy for mothers and their newborns to restore appropriate levels of this element.

In conclusion, despite the relevant guidelines, the analyzed group of pregnant women from the western Poland demonstrated an insufficient iodine status, which may present potential negative implications for pregnancy and child development. Considering that this issue is preventable, additional measures are essential in order to provide a more comprehensive information to attending physicians and medical caregivers, as well as to the general public, including young women, and to improve the iodine intake in pregnancy to the level which safely allows to avoid iodine deficiency.

## Data availability statement

The raw data supporting the conclusions of this article will be made available by the authors, without undue reservation.

## Ethics statement

The studies involving human participants were reviewed and approved by The Local Bioethics Committee of Poznan University of Medical Sciences (protocol no. 104/19, date of approval: 10 January 2019, annexed 4 February 2021, protocol no. 132/21). The patients/participants provided their written informed consent to participate in this study.

## Author contributions

Conceptualization, MR, DF, ES-P. Methodology, DF, FG, MK-Ł, AM-S, KS. Statistical analysis, DF. Investigation, DF, MR, ES-P. Data collection, DF, KS, MO. Writing - original draft preparation, DF. Writing - review and editing, MR, ES-P, LS, KS, FG, MK-Ł, AM-S, MO. Supervision, MR, ES-P. Funding acquisition, DF, MR. All authors contributed to the article and approved the submitted version.
